# Pedigree and genomic evaluation of pigs using a terminal-cross model

**DOI:** 10.1186/s12711-016-0211-3

**Published:** 2016-04-07

**Authors:** Llibertat Tusell, Hélène Gilbert, Juliette Riquet, Marie-José Mercat, Andres Legarra, Catherine Larzul

**Affiliations:** GenPhySE, Université de Toulouse, INRA, INPT, INP-ENVT, Castanet Tolosan, France; IFIP – Institut du porc, La Motte au Vicomte, BP 35104, 35651 Le Rheu Cedex, France

## Abstract

**Background:**

In crossbreeding schemes, within-line selection of purebreds is performed mainly to improve the performance of crossbred descendants under field conditions. The genetic correlation between purebred and crossbred performance is an important parameter to be assessed because purebred performance can be a poor predictor of the performance of crossbred offspring. With the availability of high-density markers, the feasibility of using crossbred information to evaluate purebred candidates can be reassessed. This study implements and applies a single-step terminal-cross model (GEN) to real data to estimate the genetic parameters of several production and quality traits in pigs.

**Methods:**

Piétrain sires were mated with Piétrain and Large White dams to produce purebred and crossbred male half-sib piglets; growth rate, feed conversion ratio, lean meat, pH of *longissimus dorsi* muscle, drip loss and intramuscular fat content were recorded on all half-sibs. Animals were genotyped using the Illumina Porcine SNP60 BeadChip. The genetic correlation between purebred and crossbred performance was estimated separately for each trait. Purebred animals were evaluated using an animal model, whereas the additive genetic effect of a crossbred individual was decomposed into the additive effects of the sire and dam and a Mendelian sampling effect that was confounded with the residual effect. Genotypes of the Piétrain animals were integrated in the genetic evaluation by using a single-step procedure. As benchmarks, we used a model that was identical to GEN but only accounted for pedigree information (PED) and also two univariate single-step models (GEN_UNI) that took either purebred or crossbred performance into account.

**Results:**

Genetic correlations between purebred and crossbred performance were high and positive for all traits (>0.69). Accuracies of estimated breeding values of genotyped sires and purebred offspring that were obtained with the GEN model outperformed both those obtained with the PED and the GEN_UNI models. The use of genomic information increased the predictive ability of the GEN model, but it did not substantially outperform the GEN_UNI models.

**Conclusions:**

We present a single-step terminal-cross model that integrates genomic information of purebred and crossbred performance by using available software. It improves the theoretical accuracy of genetic evaluations in breeding programs that are based on crossbreeding.

## Background

The use of crossbreeding in breeding schemes reduces within-line inbreeding, and the resulting heterosis and breed complementarity have favorable effects on traits of commercial interest in crossbred (CB) animals. In crossbreeding schemes, individuals from the purebred (PB) parental lines are commonly selected in highly-sanitized environments in order to improve the performance of their CB descendants under field conditions, as a correlated response. In some cases, due to genetic and environmental differences between nucleus and field conditions, the performance of nucleus PB animals can be a poor predictor of the field performance of their CB descendants, which reduces the efficiency of the breeding program in terms of genetic progress at the commercial level [1]. Thus, it is important to determine the nature of the genetic correlation between PB and CB performance and their corresponding heritabilities to assess the interest of using CB information to evaluate PB animals for CB performance [2].

The theory on which selection for CB performance is based was largely developed and discussed many years ago [3], but until now, the use of CB information to evaluate the PB parental lines has not resulted in a clear advantage for within-line PB selection and has not been widely implemented in practice. This is primarily due to practical difficulties in tracing CB pedigrees and performances back to their PB parents. However, with the availability of high-density single nucleotide polymorphism (SNP) genotype data, this scenario now needs to be reevaluated. The use of genomic information can improve response to selection by increasing the accuracy of genetic evaluations, increasing the number of candidates for selection, performing genetic evaluations at an earlier age than with traditional approaches, lowering the rate of inbreeding, avoiding the need for pedigree information to connect PB and CB individuals (depending on the method used), and accommodating non-additive genetic effects that can impact CB performance (i.e. dominance and heterosis) [4, 5].

In this study, we developed and tested on a real pig dataset a single-step terminal-cross model to estimate the genetic parameters of several production and quality traits. This model is based on the model of Wei and van der Werf [6], with an extension to include PB genotypes. The theoretical accuracies of the estimated breeding values from the mixed model equations, a pedigree-based terminal cross model, and two univariate single-step models for PB and CB performances are provided. Predictive ability of the models is also evaluated in cross-validation.

## Methods

### Animals and data

The experiment was conducted according to the French guidelines for animal care and use (http://ethique.ipbs.fr/sdv/charteexpeanimale.pdf).

Animals were produced by the four French breeding companies of the Bioporc group (ADN, Choice Genetics France, Gène+, Nucléus) involved in the UtOpIGe project ANR-10-GENOM_BTV-015, which aims at investigating the feasibility of implementing genomic selection in pyramidal breeding schemes. A large number of traits that are difficult to measure under field conditions were recorded in several PB and CB populations that were raised in the same environment. In a first step, PB Piétrain and CB Piétrain × Large White animals were produced on selection and multiplication farms and tested at a single test station. In a second step, PB Piétrain sires were mated with sows of various CB and PB populations in order to test pigs that represent usual commercial production crosses at the same test station and to validate the results obtained from the first step.

The present analyses involved only animals generated in the first step of the project, including 90 Piétrain boars (the offspring of 69 sires) and their descendants: 654 PB Piétrain and 716 CB Piétrain × Large White entire male piglets. Piétrain pigs are renowned for their very high yield of lean meat, whereas Large White pigs are reputed for their excellent maternal instinct, large litter size and high milk production. The PB and the CB descendants entered the test station facilities of Le Rheu (France) at approximately 5 weeks of age and were slaughtered at a fixed weight of 110 kg (at 5–6 months of age).

The following traits were recorded: average daily gain from the beginning (35 kg) to the end (110 kg) of the test period (ADG), feed conversion ratio (FCR), % lean meat (LM), pH of the *longissimus dorsi* muscle (pH), drip loss (DL), and intramuscular fat (IMF). Data were obtained in accordance with the national regulations on the welfare of animals used in research.

At the slaughterhouse, carcasses were chilled in a cooling room at 4 °C for 24 h and right half-carcasses were cut [7]. LM was estimated from a linear combination of the weights of cuts that were expressed as a percentage of the cold half-carcass weight for ham, loin and backfat [8]. Ultimate pH of the *longissimus dorsi* muscle was measured using a Xerolyt electrode (Mettler-Toledo, Australia) and a Sydel pH meter (Sydel, France). DL was measured on a sample of loin of about 130 g (at the 13th lumbar vertebra). After weighing the samples, they were placed directly in a polystyrene tray, covered with polyethylene film and stored at 4 °C for 48 h, such that each slice formed a 40-degree angle with the horizontal plane. Then, the samples were wiped gently and weighed again. The DL was quantified as the difference between the two weights, expressed as a percentage of the initial weight. After DL measurement, the samples were frozen until IMF was measured by magnetic resonance imaging [9].

A separate pedigree file was constructed for each PB line. Piétrain and Large White pedigrees were constructed up to five generations back from the Piétrain boars for which data were available as PB or sires of CB offspring and from the Large White sows for which data were available as dams of CB offspring, respectively.

The 90 Pietrain boars and their PB descendants were genotyped using the Illumina Porcine SNP60 BeadChip (Illumina, Inc., San Diego). SNPs with a call rate lower than 0.90 and a minor allele frequency lower than 0.05 were removed. For the remaining SNPs, the very few missing genotypes were imputed using a naïve method that sampled the genotypes with probability weights based on allele frequencies at each locus. Animals with a call rate lower than 0.90 and progeny that displayed Mendelian inconsistencies with their parents were discarded. Summary statistics of the phenotypes, pedigrees and genotypes are in Tables 1 and 2. Given that the number of animals with records differed for each trait, data were edited separately for each trait. Thus, the number of SNPs retained for the analyses differed slightly between traits.

**Table 1:** Summary statistics of the purebred/crossbred phenotype data

	Growth rate	Feed conversion ratio	Lean meat	pH *longissimus dorsi*	Drip loss	Intramuscular fat
Units	g/day	kg/kg	%	pH units	%	%
Minimum	511.1/592.6	1.83/1.67	54.8/57.7	5.29/.27	1.35/0.74	0.45/0.23
Mean	938.5/1038.0	2.29/2.25	64.05/62.48	5.58/5.62	7.26/4.87	1.14/1.21
Maximum	1214.9/1291.0	3.20/2.7	67.80/67.6	6.42/6.51	16.54/15.50	2.15/2.26
Coefficient of variation	0.11/0.09	0.07/0.07	0.02/0.03	0.03/0.03	0.38/0.40	0.21/0.21
Number of records	654/716	631/709	638/13	640/714	614/689	538/650

**Table 2:** Summary statistics of the purebred/crossbred pedigree and genotype data

	Growth rate	Feed conversion ratio	Lean meat	pH *longissimus dorsi*	Drip loss	Intramuscular fat
Number purebred offspring	654	631	638	640	614	538
Number crossbred sires/dams	90/306	90/306	90/306	90/306	90/304	89/302
Number animals in the Piétrain pedigree	3084	3036	3052	3057	3007	2/900
Number animals in the Large White pedigree	2686	2686	2686	2686	2676	2/677
Number SNPs, after editing	39,672	39,673	39,673	39,681	39,650	39,643
Number genotyped PB offspring individuals/sires, after editing	635/89	616/89	626/89	628/89	603/89	530/88

*SNP* single-nucleotide polymorphism, *PB* purebred, *CB* crossbred

### Statistical analyses

PB and CB phenotypes for a trait were considered as two different traits. They were analyzed jointly by adapting the terminal-cross model proposed by Wei and van der Werf (see Appendix 2 in [6]) by either using pedigree information only or by combining pedigree and genomic information in a single-step procedure [10–12]. The model used here is a simplification of the model of Christensen et al. [13], who considered the inclusion of CB genotypes.

#### Pedigree-based terminal-cross model

In matrix notation, the PB and CB records of a given trait in a pedigree-based terminal-cross model (PED) can be represented as follows:

1 $$ \begin{aligned} \left[ {\begin{array}{*{20}c} {{\mathbf{y}}_{A} } \\ {{\mathbf{y}}_{C} } \\ \end{array} } \right] & = \left[ {\begin{array}{*{20}c} {{\mathbf{X}}_{A} } & 0 \\ 0 & {{\mathbf{X}}_{C} } \\ \end{array} } \right]\left[ {\begin{array}{*{20}c} {{\mathbf{b}}_{A} } \\ {{\mathbf{b}}_{C} } \\ \end{array} } \right] + \left[ {\begin{array}{*{20}c} {{\mathbf{W}}_{A} } & 0 \\ 0 & {{\mathbf{W}}_{C} } \\ \end{array} } \right]\left[ {\begin{array}{*{20}c} {{\mathbf{p}}_{A} } \\ {{\mathbf{p}}_{C} } \\ \end{array} } \right] \\ & \quad + \left[ {\begin{array}{*{20}c} {{\mathbf{Z}}_{A} } & 0 & 0 \\ 0 & {{\mathbf{Z}}_{AC} } & {{\mathbf{Z}}_{BC} } \\ \end{array} } \right]\left[ {\begin{array}{*{20}c} {{\mathbf{u}}_{AA} } \\ {{\mathbf{u}}_{AC} } \\ {{\mathbf{u}}_{BC} } \\ \end{array} } \right] + \left[ {\begin{array}{*{20}c} {{\mathbf{e}}_{A} } \\ {{\mathbf{e}}_{C} } \\ \end{array} } \right], \\ \end{aligned} $$

where ${\mathbf{y}}_{k}$ is a vector of phenotypes for the PB Piétrain (for *k* = *A*,) and CB Piétrain × Large White individuals (for *k* = *C*), ${\mathbf{b}}_{k}$ is a vector of systematic effects, ${\mathbf{p}}_{k}$ is a vector of the random pen effects (nested within batch), and ${\mathbf{e}}_{k}$ is a vector of residual effects. ${\mathbf{X}}_{k}$, ${\mathbf{W}}_{k}$ and ${\mathbf{Z}}_{A}$, ${\mathbf{Z}}_{AC}$ and ${\mathbf{Z}}_{BC}$ are incidence matrices that assign systematic, pen and additive genetic effects, respectively, to the phenotypes. A brief description of the effects included in the model for each trait analyzed is in Table 3. Vector ${\mathbf{u}}_{AA}$ is the vector of additive genetic effects for the PB pigs. The additive genetic effect for the CB individuals (${\mathbf{u}}_{CC}$) is decomposed into the additive gametic effects for CB performance of their corresponding Piétrain sires and Large White dams (${\mathbf{u}}_{AC}$ and ${\mathbf{u}}_{BC}$, respectively) and the corresponding sire and dam Mendelian sampling effects ($\varvec{\phi}_{A}$ and $\varvec{\phi}_{B}$, respectively):

$$ {\mathbf{u}}_{CC} = {\mathbf{Z}}_{AC} {\mathbf{u}}_{AC} + {\mathbf{Z}}_{BC} {\mathbf{u}}_{BC} +\varvec{\phi}_{A} +\varvec{\phi}_{B} . $$

**Table 3:** Systematic and permanent environmental random effects included in the models of analysis for each trait

Effect	Trait					
	Growth rate	Feed conversion ratio	Lean meat	pH *longissimus dorsi*	Drip loss	Intramuscular fat
Weight at the beginning of the control period	Covariate	Covariate	–	–	–	–
Hot carcass weight	–	–	Covariate	–	–	–
Weight at slaughter	–	–	–	Covariate	Covariate	Covariate
Date of slaughter	–	–	–	52 levels	50 levels	49 levels
Batch	11 levels	11 levels	11 levels	11 levels	11 levels	11 levels
Pen effect nested within batch	132 levels	132 levels	132 levels	–	–	–

The Mendelian sampling effects cannot be estimated and are, therefore, included in the residual effect of the CB part of the model (Eq. 1). Note that ${\mathbf{u}}_{AA}$ and ${\mathbf{u}}_{AC}$ have the same dimension and that two genetic effects are assigned to each PB Piétrain animal.

The variance–covariance structure of additive genetic effects was assumed to follow:

$$ var\left[ {\begin{array}{*{20}c} {{\mathbf{u}}_{AA} } \\ {{\mathbf{u}}_{AC} } \\ {{\mathbf{u}}_{BC} } \\ \end{array} } \right] = \left[ {\begin{array}{*{20}c} {{\mathbf{A}}_{A} \sigma_{A}^{2} } & {{\mathbf{A}}_{A} \sigma_{{A\left( {AC} \right)}} } & 0 \\ {{\mathbf{A}}_{A} \sigma_{{A\left( {AC} \right)}} } & {{\mathbf{A}}_{A} \sigma_{AC}^{2} } & 0 \\ 0 & 0 & {{\mathbf{A}}_{B} \sigma_{BC}^{2} } \\ \end{array} } \right], $$

where ${\mathbf{A}}_{A}$ and ${\mathbf{A}}_{B}$ are the relationship matrices for the Piétrain and the Large White individuals, respectively, computed based on their corresponding pedigrees. $\sigma_{A}^{2}$ is the additive genetic variance of the Piétrain line for PB performance, $\sigma_{AC}^{2}$ and $\sigma_{BC}^{2}$ are the additive genetic variances of the additive genetic contributions of Piétrain and Large White breeds, respectively, to CB performance, which are equal to one quarter of the genetic variance in a traditional sense (see below). Finally, $\sigma_{{A\left( {AC} \right)}}^{  }$ is the additive genetic covariance between PB and CB performance of the Piétrain animals. For the maternal line, only the additive genetic variance for CB performance was estimated. There were no correlations between pen effects, $$ {\mathbf{P}} = diag({\begin{array}{*{20}c} {\sigma_{pA}^{2} } & {\sigma_{pC}^{2} } \\ \end{array} }) $$ and between residual effects, $$ {\mathbf{R}} = diag( {\begin{array}{*{20}c} {\sigma_{eA}^{2} } & {\sigma_{eC}^{2} } \\ \end{array} } ) $$, or between these effects and other random effects.

#### Single-step terminal-cross model

The form of the single-step terminal-cross model (GEN) is the same as that of Eq. (1). In the GEN model, the inverse of the numerator relationship matrix of the Piétrain line (${\mathbf{A}}_{A}^{ - 1}$) that was used in the mixed model equations to estimate the breeding values [14] is replaced by the inverse of the ${\mathbf{H}}$ matrix (${\mathbf{H}}^{ - 1}$) that combines both the genomic (${\mathbf{G}}$) and the pedigree-based relationship matrices allowing the joint genetic evaluation of genotyped and non-genotyped animals [12, 15]:

$$ {\mathbf{H}}^{ - 1} = {\mathbf{A}}_{\varvec{A}}^{ - 1} + \left[ {\begin{array}{*{20}c} 0 & 0 \\ 0 & {{\mathbf{G}}^{ - 1} - {\mathbf{A}}_{{\left( \varvec{A} \right)22}}^{ - 1} } \\ \end{array} } \right], $$

where ${\mathbf{G}}_{ }^{ - 1}$ is the inverse of the genomic relationship matrix and ${\mathbf{A}}_{{\left( \varvec{A} \right)22}}^{ - 1}$ is the inverse of the pedigree-based relationship matrix of the genotyped animals.

For PB Piétrain individuals (i.e. PB descendants and their sires), ${\mathbf{G}}$ was calculated following the default single-step procedure as programmed in blupf90 (http://nce.ads.uga.edu/wiki/lib/exe/fetch.php?media=blupf90_all1.pdf). First, Van Raden’s equation [16] was used:

$$ {\mathbf{G}}^{*} = \frac{{\left( {{\mathbf{X}} - {\mathbf{E}}} \right)\left( {{\mathbf{X}} - {\mathbf{E}}} \right)^{'} }}{{2\mathop \sum \nolimits_{j = 1}^{p} q_{j} \left( {1 - q_{j} } \right)}}, $$

where SNP genotypes were coded as 0, 1, and 2 for animals that were homozygous for the minor allele, heterozygous, and homozygous for the other allele, respectively. This leads to ${\mathbf{X}} = \left\{ {x_{ij} } \right\}$, a matrix of dimension *n* × *p* (*i* = 1, …, *n* and *j* = 1, …, *p,* with *n* being the number of genotyped individuals and *p* the number of SNPs), *q*_*j*_ is the frequency of the minor allele of the *j*th SNP. Each column of matrix ${\mathbf{E}}$ contains twice the expected genotype frequencies at each locus. Given that the allele frequencies from the base population under Hardy–Weinberg equilibrium were not available, the allele frequencies among the genotyped animals were used to calculate the expected genotype frequencies.

Then, ${\mathbf{G}}^{\varvec{*}}$ was adjusted to match the average level of inbreeding and coancestries of ${\mathbf{A}}_{\left( A \right)22}$, as described by Christensen et al. [17]. Finally, ${\mathbf{G}} = 0.95\;{\mathbf{G}}^{\varvec{*}} + 0.05\;{\mathbf{A}}_{\left( A \right)22}$ to make ${\mathbf{G}}$ invertible. Similar to the PED model, the GEN model used only pedigree information (${\mathbf{A}}_{B}$) to estimate $\upsigma_{BC}^{2}$.

#### Single-step univariate models

Single-step univariate models (GEN_UNI) were also run separately for PB and CB phenotypes by including the same effects as for the GEN model:

$$ {\mathbf{y}}_{A} = {\mathbf{X}}_{A} {\mathbf{b}}_{A} + {\mathbf{W}}_{A} {\mathbf{p}}_{A} + {\mathbf{Z}}_{A} {\mathbf{u}}_{AA} + {\mathbf{e}}_{A} , $$

$$ {\mathbf{y}}_{C} = {\mathbf{X}}_{C} {\mathbf{b}}_{C} + {\mathbf{W}}_{C} {\mathbf{p}}_{C} + {\mathbf{Z}}_{AC} {\mathbf{u}}_{AC} + {\mathbf{Z}}_{BC} {\mathbf{u}}_{BC} + {\mathbf{e}}_{C} . $$

### Parameter inference

A Bayesian framework was adopted for inference to express uncertainty about the unknowns through the use of probability density functions. Flat prior distributions were assumed for the parameters of the systematic effects and the (co)variance components. The Gibbs sampler algorithm was used to estimate the marginal posterior distributions of the systematic effects and the (co)variance components using the GIBBS1f90 software developed by Misztal et al. [18]. Single chains of 250,000, 500,000 and 250,000 iterations were run by discarding the first 25,000, 50,000 and 25,000 iterations of each chain for the PED, GEN and GEN_UNI models, respectively, for each analyzed trait. Longer chains were run for the GEN models due to the less sparse structure of the single-step equations, which may preclude good mixing of the chains. The number of discarded samples was, in all cases, larger than the required burn-in that was determined by visual inspection and by the procedures of Raftery and Lewis [19] and Geweke [20]. Samples of the parameters of interest were saved every ten rounds and used to compute summary statistics for the marginal posterior distributions.

### Rescaling additive genetic contributions of sires and dams to the crossbred trait

Because CB offspring performance includes only half of the breeding values for CB performance of their PB parents, Wei and van der Werf [6] assigned 0.5 instead of 1 to the non-zero elements of the design matrices ${\mathbf{Z}}_{AC}$ and ${\mathbf{Z}}_{BC}$ in Eq. (1). In our case, we assigned ones to the non-zero elements of the incidence matrices for practical implementation purposes. Thus, posterior rescaling of the genetic variances was required to obtain proper (co)variance estimates. For example on the paternal side, ${\mathbf{u}}_{AC}$ is equal to half the additive genetic effect of the sire for CB performance. To recover the corresponding paternal breeding value, each gametic contribution was multiplied by 2, i.e. ${\mathbf{u}}_{AC}^{*} = 2{\mathbf{u}}_{AC}$. This, in turn, leads to rescaling the parental genetic variance as $var({\mathbf{u}}_{AC}^{*} ) = 4\;var\left( {{\mathbf{u}}_{AC} } \right) = 4\sigma_{AC}^{2}$ and the additive genetic covariance as *σ*_*A*(*AC*)_^*^ = 2*σ*_*A*(*AC*)_. The same rescaling also applies for the genetic variance on the maternal side.

### Theoretical accuracies of estimated breeding values

The posterior mean (co)variance components obtained with the Gibbs sampler for the GEN model were used as the true values to obtain best linear unbiased prediction (BLUP) estimates for the breeding values in the different models using the BLUPf90 software [18]. The theoretical accuracy of the estimated breeding value for the *i*th individual for the *k*th (*k* = *A*, *AC*) performance with the *m*th model ($m = GEN, PED, GEN\_UNI$) (*EBV*_*i*,*k*,*m*_) was calculated as [13]:

$$ R_{i,k,m} = \sqrt {1 - \frac{{PEV_{i,k,m} }}{{\left( {1 + F_{i} } \right)\sigma_{k}^{2} }}} , $$

where *PEV*_*i*,*k*,*m*_ is the prediction error variance of its *EBV*_*i*,*k*,*m*_, *F*_*i*_ is the inbreeding coefficient of individual *i*, which was computed from the pedigree using the INBUPGf90 software [21], and *σ*_*k*_^2^ is the additive genetic variance of PB or CB performance. Theoretical accuracies of EBV were obtained for all sires and PB offspring and also for some PB descendants that were considered to be candidates for selection (one descendant if the sire had less than four male offspring and two otherwise). Theoretical accuracies of EBV for these candidates were obtained using BLUP by either masking or including their own phenotype, in order to reproduce a situation under commercial conditions where some traits are measured on candidates and other traits are measured on relatives that are housed in test stations.

### Assessment of predictive ability

The ability of the models to predict yet-to-be observed phenotypes was compared using sixfold cross-validation. First, BLUP solution estimates for the systematic effects obtained with the GEN model were used to obtain adjusted phenotype records, separately for each trait. Second, sires were randomly split into six approximately equal subsets. All records of the offspring of a sire were assigned to its respective subset. BLUP parameters were estimated based on five of the six data subsets, referred to as the training set, and the predictive ability was assessed in the remaining dataset, which was used as the testing set and considered as the yet-to-be observed phenotypes of the candidate sires. The training–testing cycle was repeated six times by rotating the six subsets used for training and testing, with each subset used only once for testing and five times for training. The predictive ability of each model was evaluated using the average Pearson’s correlation between observed and predicted phenotypes of the testing sets.

In addition, the EBV of sires for PB performance estimated with the GEN_UNI model were used to predict CB performance under the GEN_UNI CB model. This predictive ability was also assessed with the sixfold cross-validation mentioned above.

## Results and discussion

### Genetic parameters

Genetic parameter estimates, ratios of variance components, and ratios of phenotypic variances obtained with the GEN model are in Table 4. Most of the estimated heritabilities for traits related to PB performance were within the range of those obtained in previous studies on pigs [22–25], although an unusually high value was found for drip loss, i.e. 0.57 compared to published values, which range from 0.10 to 0.30 [26].

**Table 4:** Mean (highest posterior density interval at 95 %) of the marginal posterior distribution of genetic parameters estimated with the single-step model

Trait	Parameter							
	*h*_*A*_^2^	*t*_*AC*_^2^	*t*_*BC*_^2^	*rg*_*A*,* AC*_	*p*_*A*_^2^	*p*_*C*_^2^	*σ*_*A*_^2^	*σ*_*C*_^2^
Growth rate	0.22 [0.05, 0.37]	0.25 [0.03, 0.45]	0.28 [0.12, 0.44]	0.84 [0.45, 1.00]	0.14 [0.06, 0.23]	0.09 [0.02, 0.16]	9151 [7954, 10,418]	8028 [6813, 9324]
Feed conversion ratio	0.32 [0.20, 0.46]	0.29 [0.13, 0.46]	0.18 [0.05, 0.32]	0.91 [0.72, 1.00]	0.10 [0.03, 0.18]	0.09 [0.03, 0.16]	0.02 [0.02, 0.03]	0.02 [0.02, 0.02]
Lean meat	0.41 [0.25, 0.57]	0.30 [0.14, 0.46]	0.28 [0.12, 0.45]	0.69 [0.30, 1.00]	0.04 [0.0001, 0.08]	0.06 [0.01, 0.12]	2.50 [2.19, 2.82]	3.15 [2.75, 3.59]
pH *longissimus dorsi*	0.30 [0.17, 0.36]	0.26 [0.16, 0.40]	0.11 [0.02, 0.22]	0.97 [0.83, 1.00]	–	–	0.02 [0.02, 0.02]	0.02 [0.02, 0.03]
Drip loss	0.57 [0.44, 0.69]	0.21 [0.08, 0.35]	0.15 [0.03, 0.30]	0.89 [0.62, 1.00]	–	–	6.21 [5.41, 7.04]	3.38 [2.96, 3.81]
Intramuscular fat	0.31 [0.16, 0.48]	0.34 [0.16, 0.54]	0.25 [0.08, 0.43]	0.82 [0.46, 1.00]	–	–	0.05 [0.05, 0.06]	0.07 [0.06, 0.08]

*h*_*A*_^2^ = purebred heritability

*t*_*iC*_^2^ = ratio of variance of the parental allelic contribution in the crossbreds (*i* = *A*, *B* for Piétrain and Large White line, respectively) computed as 2*σ*_*iC*_^2^/*σ*_*C*_^2^ where *σ*_*iC*_^2^ is the additive genetic variance of the corresponding parental alleles in the crossbreds

*rg*_*A*, *AC*_ = genetic correlation between purebred individual and sire line contribution in the crossbreds

*p*_*j*_^2^ = ratio of variance of common pen effect

*σ*_*j*_^2^ = phenotypic variances (*j* = *A*, *C* for purebred Piétrain and crossbred, respectively)

Estimated genetic correlations between PB Piétrain and CB performance of Piétrain sires were all positive and high and none of the highest 95 % posterior density intervals (HPD_95 %_) of the estimates included values below 0.30. This indicates that most of the genetic variance observed for those traits is due to additive genes with no relevant dominance gene action and, possibly, no differences in gene frequency between the two lines, i.e. there is no strong genetic interaction between the Piétrain and Large White breeds. The magnitude and sign of the estimated genetic correlation between PB and CB performance are keys to decide the best strategy to evaluate PB animals for CB performance [2]. Hence, based on our results and the conditions under which this study was performed, selecting to improve traits within the paternal PB line, without accounting for CB information, would lead to an improvement in the CB population as a correlated response [27]. Whether the degree of this improvement would overcome the gain of incorporating CB performance needs to be further addressed. Various ranges of genetic correlation estimates between PB and CB performances have been reported in the literature using different pedigree-based approaches. Apart from a few exceptions, they range from moderate to high values for production traits such as lifetime daily gain, feed conversion ratio, back fat thickness, and weight (see reviews in [1, 28, 29]. For genetic correlations that differ from 1, other selection strategies might be more appropriate to improve genetic response in CB descendants, for example: (1) evaluating the PB lines based on CB information only (recurrent selection and reciprocal recurrent selection [3]), (2) combining both PB and CB information into a weighted selection index [30, 31], (3) using a terminal-cross model [6, 13], or (4) using a multiple-trait approach with one additive effect [32], although the latter can lead to biased estimates of PB CB covariance [33]. Genetic correlations between PB and CB performances can differ from 1 if non-additive genetic effects, such as dominance are present and allele frequencies differ between the parental lines [34]. Genetic effects can also vary with the environment in which PB and CB individuals are raised, which can also contribute to genetic correlations differing from 1. Such situations are common in pig breeding, with PB lines reared and evaluated on nucleus farms that are defined by a high health status environment and CB pigs raised on commercial farms under field conditions. In the current study, all animals were raised at the same time and in the same test station facility and differed only in the genetic origin of the dams. Thus, the environment was simply defined by the breeding type [35].

The ratios of genetic variance for CB performance for the sire and dam lines were of similar magnitude across the traits, although they tended to be slightly higher for the sire line for FCR, pH and IMF (Table 4). Heritabilities for CB performance are the sum of the ratios of paternal and maternal line genetic contributions (Table 4, $t_{\text{AC}}^{2} + t_{\text{BC}}^{2}$) and were approximately of the same magnitude as heritabilities for PB performance for FCR and pH, but were higher for ADG, LM and IMF, and lower for DL. Some studies have reported lower heritabilities for CB than for PB performance, which is mainly due to a less controlled environment for the CB field data compared with the PB station data [36] but this does not apply to our study.

The additive genetic variances (*h*_*A*_^2^*σ*_*A*_^2^ and *t*_*AC*_^2^*σ*_*C*_^2^ + *t*_*BC*_^2^*σ*_*C*_^2^ for PB and CB performance, respectively; Table 4) were slightly higher for crossbred than for PB performance for most of the analyzed traits, which could indicate subtle differences in gene combinations affecting the traits in the two populations, and a slightly stronger influence of non-additive genetic effects, such as dominance, in CB than in PB individuals. Under dominance action, the additive genetic variance of CB individuals cannot be predicted by calculating the average of the additive genetic variances of the parental lines for purebred performance and it can be larger than either of the parental genetic variances [2]. In another study that applied several genome-enabled prediction models, Tusell et al. [37] found that the estimated additive genetic variance and heritability for litter size were higher in a CB population of commercial pigs than in either of the PB parental lines, and suggested that it could be due to a lower level of heterozygosity of PB sows compared to CB sows. In contrast, Lutaaya et al. [29] reported a smaller additive genetic variance for backfat in a CB pig line than in the PB parental lines. They attributed this result to differences in management practices, reduced genetic variation due to the fact that the selection index previously used for CB parents included gain and carcass traits, and to differences in sex ratios between the PB and CB populations, since most of the CB individuals were females.

Given the magnitude of the estimates of heritabilities and genetic correlations between PB and CB performances, the allelic frequencies between the two breeds seem to be similar and the analyzed traits do not appear to be affected by non-additive genetic effects. Regardless, the model presented here is somehow able to capture the general level of heterosis of each line into the general mean effect of each trait [13]. It would be of interest to extend this single-step terminal cross model to account for dominance effects for the analysis of traits that are more affected by non-additive genetic effects.

### GEN versus PED model

The posterior mean estimates of heritability for PB performance obtained with the PED model for the different traits (Table 5) were slightly higher than those obtained with the GEN model (Table 4). This is due to the non-normal posterior distribution of this parameter estimate with the PED model, which was more right-skewed than for the GEN model, and the lower precision that was obtained, which resulted in higher posterior mean estimates than the GEN model; frequency histograms showed that the posterior modes of this parameter were very similar between the two models. The GEN model provided more precise estimates, possibly due to the greater amount of information used, i.e. it combined genome-based relationships together with pedigree-based relationships.

**Table 5:** Mean (highest posterior density interval at 95 %) of the marginal distribution of genetic parameter estimated with the pedigree-based model

Trait	Parameter							
	*h*_*A*_^2^	*t*_*AC*_^2^	*t*_*BC*_^2^	*rg*_*A*,* AC*_	*p*_*A*_^2^	*p*_*C*_^2^	*σ*_*A*_^2^	*σ*_*C*_^2^
Growth rate	0.33 [0.08, 0.57]	0.24 [0.11, 0.40]	0.29 [0.12, 0.44]	0.79 [0.37, 1.00]	0.13 [0.04, 0.22]	0.09 [0.03, 0.15]	9239 [7968.70, 1, 0550.00]	8032 [7001.20, 9153.20]
Feed conversion ratio	0.37 [0.21, 0.56]	0.29 [0.14, 0.45]	0.18 [0.06, 0.32]	0.89 [0.66, 1.00]	0.10 [0.03, 0.18]	0.09 [0.03, 0.16]	0.02 [0.02, 0.03]	0.02 [0.02, 0.02]
Lean meat	0.46 [0.24, 0.67]	0.28 [0.13, 0.45]	0.29 [0.13, 0.46]	0.74 [0.34, 1.00]	0.04 [0.001, 0.09]	0.06 [0.01, 0.12]	2.51 [2.20, 2.82]	3.13 [2.75, 3.56]
pH *longissimus dorsi*	0.46 [0.22, 0.76]	0.27 [0.13, 0.39]	0.11 [0.02, 0.22]	0.91 [0.57, 1.00]	–	–	0.02 [0.01, 0.02]	0.02 [0.02, 0.03]
Drip loss	0.70 [0.52, 0.89]	0.22 [0.09, 0.38]	0.15 [0.03, 0.28]	0.87 [0.58, 1.00]	–	–	6.30 [5.52, 7.20]	3.39 [2.98, 3.85]
Intramuscular fat	0.40 [0.18, 0.62]	0.34 [0.17, 0.52]	0.25 [0.09, 0.40]	0.86 [0.56, 1.00]	–	–	0.05 [0.05, 0.06]	0.07 [0.06, 0.08]

*h*_*A*_^2^ = purebred heritability

*t*_*iC*_^2^ = ratio of variance of the parental allelic contribution in the crossbreds (*i* = *A*, *B* for Piétrain and Large White line, respectively) computed as 2*σ*_*iC*_^2^/*σ*_*C*_^2^ where *σ*_*iC*_^2^ is the additive genetic variance of the corresponding parental alleles in the crossbreds

*rg*_*A*,* AC*_ = genetic correlation between purebred individual and sire line contribution in the crossbreds

*p*_*j*_^2^ = ratio of variance of common pen effect

*σ*_*j*_^2^ = phenotypic variances (*j* = *A*, *C* for purebred Piétrain and crossbred, respectively)

Table 6 shows the mean accuracies of EBV obtained with the GEN model for PB and CB performance of the genotyped animals, i.e. the PB offspring and their sires, and the mean difference of these accuracies from the PED model. For all traits, EBV accuracies were higher for the GEN model than for the PED model because, to estimate EBV, the GEN model uses more information than the PED model, as explained above. Several studies have found accuracies for the EBV of genotyped animals to be higher when genomic information is included in the models compared to using pedigree data only [38, 39]. In contrast, the mean difference in accuracies of EBV between GEN and PED models for animals in the pedigree without own records and genotypes was almost equal to 0 and ranged from 0.002 to 0.008 for both PB and CB performance. The same results were observed for the dams of the CB offspring because no extra information was used to estimate the EBV of these animals in the GEN model.

**Table 6:** Mean (SD) accuracy of EBV for purebred and crossbred performance obtained using single-step terminal-cross models and its difference (SD) from the mean accuracy obtained using pedigree-based terminal-cross models

Trait	PB performance				CB performance			
	PB offspring		Sires		PB offspring		Sires	
	Mean	Mean difference	Mean	Mean difference	Mean	Mean difference	Mean	Mean difference
ADG	0.577 (0.026)	0.050 (0.021)	0.660 (0.040)	0.039 (0.016)	0.514 (0.033)	0.042 (0.027)	0.697 (0.047)	0.022 (0.012)
FCR	0.660 (0.020)	0.044 (0.015)	0.732 (0.036)	0.034 (0.012)	0.615 (0.023)	0.041 (0.018)	0.745 (0.040)	0.024 (0.010)
LM	0.699 (0.016)	0.039 (0.013)	0.716 (0.040)	0.047 (0.016)	0.540 (0.030)	0.032 (0.024)	0.724 (0.044)	0.019 (0.010)
pH	0.639 (0.024)	0.046 (0.018)	0.745 (0.039)	0.027 (0.011)	0.624 (0.025)	0.045 (0.018)	0.750 (0.040)	0.025 (0.011)
DL	0.768 (0.020)	0.030 (0.012)	0.768 (0.040)	0.044 (0.015)	0.680 (0.020)	0.026 (0.015)	0.741 (0.039)	0.030 (0.012)
IMF	0.626 (0.029)	0.042 (0.018)	0.688 (0.047)	0.034 (0.015)	0.548 (0.031)	0.038 (0.023)	0.730 (0.052)	0.018 (0.010)

*PB* purebred, *CB* crossbred, *ADG* growth rate between end and beginning of the control period, *FCR* Feed conversion ratio, *LM* % of lean meat, *pH* pH *longissimus dorsi*, *DL* drip loss, *IMF* intramuscular fat

### Theoretical accuracies of EBV from the GEN model

**Fig. 1:**
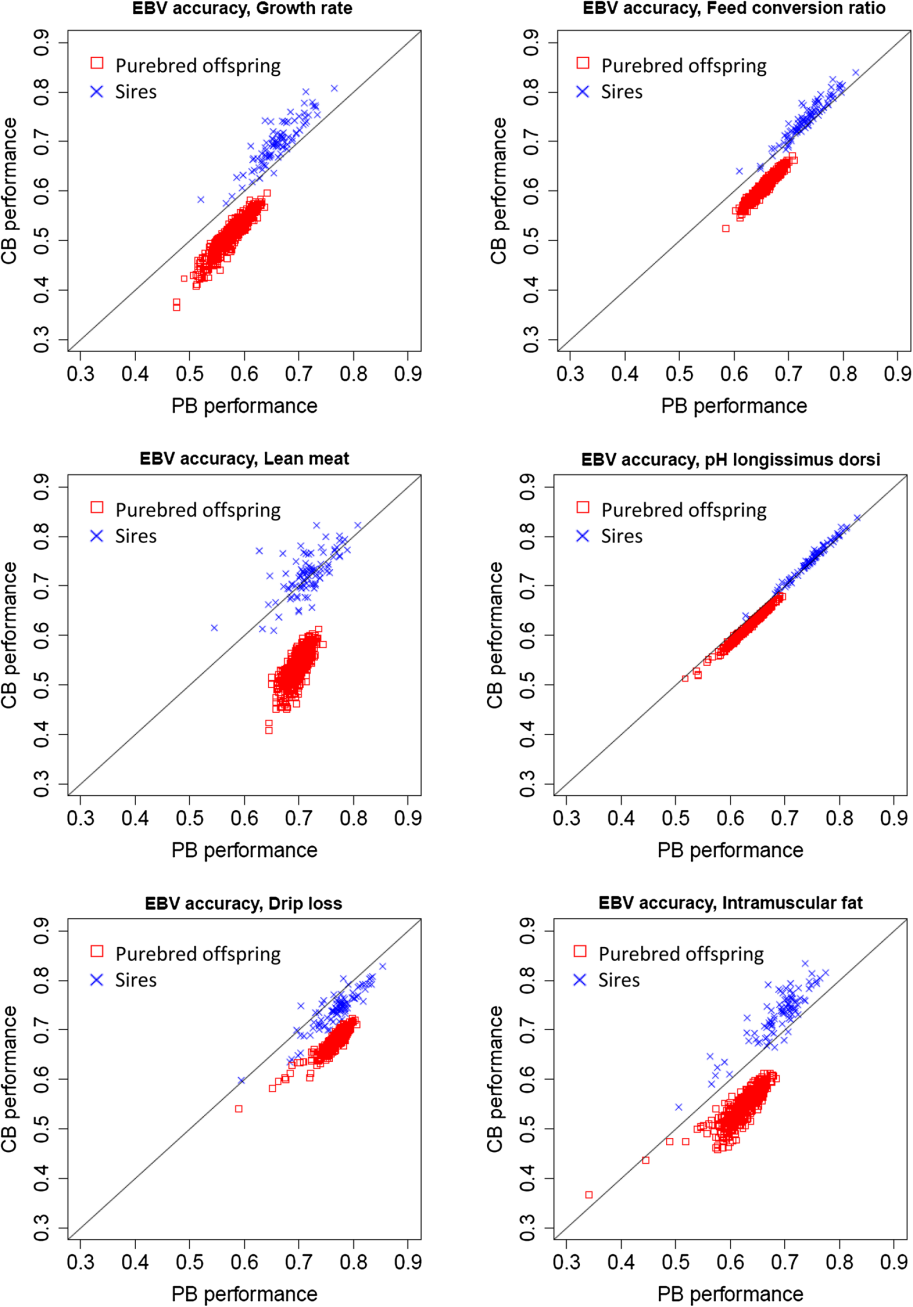
*Scatterplots* of the accuracies of estimated breeding values for purebred versus crossbred performance obtained with single-step terminal-cross models. *EBV* estimated breeding value, *PB* purebred, *CB* crossbred

Scatterplots of the theoretical accuracies of EBV for PB performance versus those for CB performance of sires and PB offspring obtained with the GEN model for the different traits are in Fig. 1. Accuracies were higher for the sires than for the PB offspring for both PB and CB performance. This may be due to the fact that among all evaluated individuals, the sires have the largest amount of information available for both traits because they are sires of both PB and CB offspring. As expected, the accuracies of EBV were higher when the animals had records for either PB or CB performance. For the sires, this was because their EBV for CB performance in a terminal-cross model is estimated directly through the sire genetic effect, whereas the EBV of the same sire evaluated for PB performance is estimated through the animal genetic effect of their PB offspring. For the PB offspring, accuracies of their EBV were much higher when evaluated for PB than for CB performance. This is because the EBV for CB performance of the PB offspring was estimated based only on records on their sires and half-sibs. This increase was more pronounced for traits such as ADG and IMF and less for pH.

### Accuracies of EBV from the GEN_UNI model

**Fig. 2:**
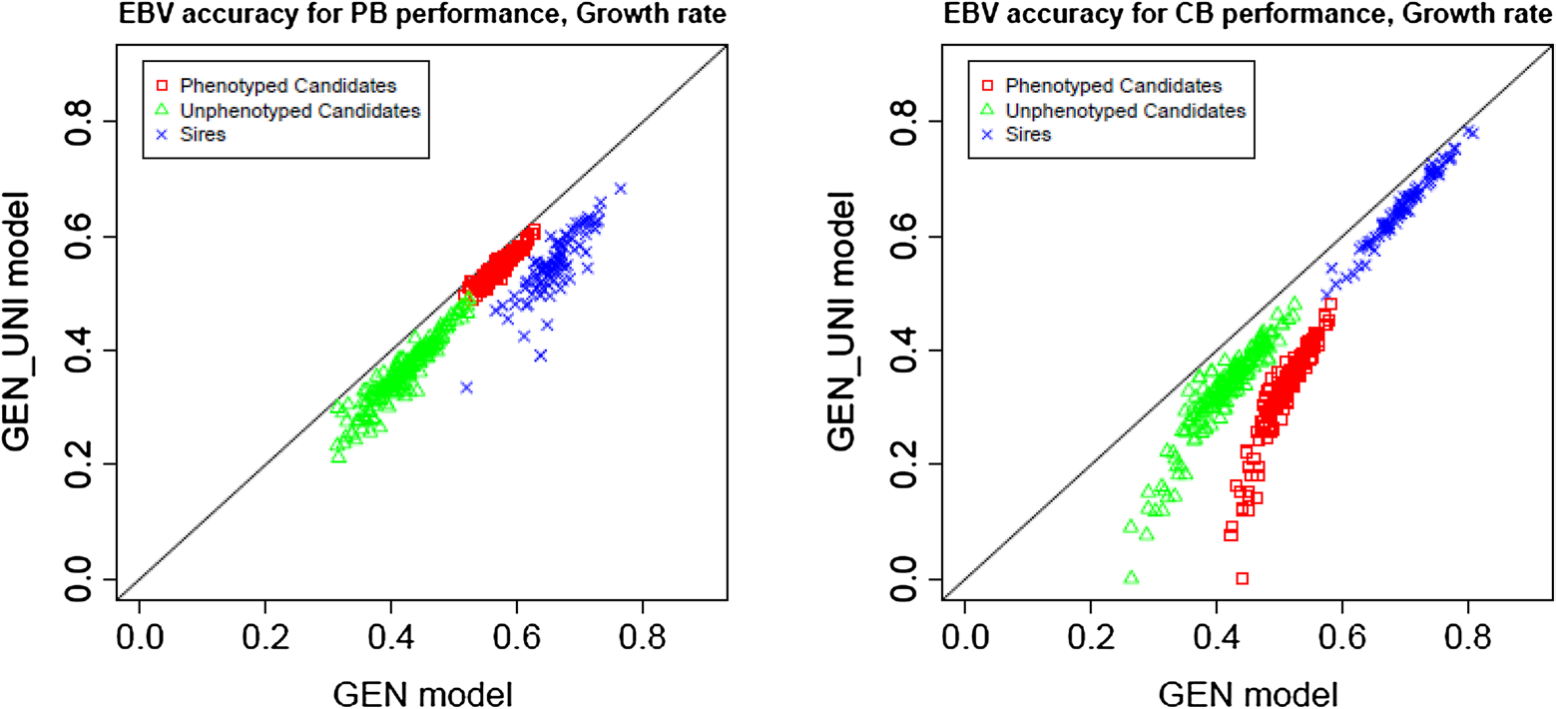
*Scatterplots* of estimated breeding values obtained from a single-step terminal-cross model versus those from a univariate single-step models for growth rate. *EBV* estimated breeding value, *CB* crossbred, *PB* purebred, *GEN* single-step terminal-cross model, *GEN_UNI* univariate single-step models for purebred or crossbred performance

The mean theoretical accuracies of the EBV of sires and candidates to selection (i.e. one or two PB offspring per sire with and without own phenotype) obtained with the single-step terminal-cross model and the mean differences of these accuracies from those obtained with the two univariate single-step models (one for CB and the other for PB performance) across traits are in Table 7. Figure 2 shows scatterplots of the EBV accuracies obtained with the GEN model versus those obtained with the GEN_UNI models for sires and candidates to selection for PB and CB performance for ADG; the other traits followed a very similar pattern (not shown). Accuracies of the EBV for sires were higher when obtained with the single-step terminal-cross model than with the univariate single-step models for both PB and CB performance. Hence, accounting for PB and CB information in a two-trait model enhances the theoretical accuracy of EBV of sires for PB and CB performances. This is possibly due to the more precise variance component estimates, i.e. narrower HPD95 %, obtained with the GEN model compared to the GEN_UNI models (not shown). Lutaaya et al. [40] reported a higher reliability of crossbred EBV of purebred animals when a terminal-cross model was used compared to a within-line model due to smaller standard errors of the variance component estimates. They stated that the terminal-cross model was more reliable because it uses all the information that is available on the progeny. Lutaaya et al. [40] highlighted the advantage of using a terminal-cross model under two scenarios: first, when EBV for both PB and CB evaluation performances are of interest and a sufficient number of CB records is available, and second when some traits are recorded on PB animals whereas others are recorded only on CB animals. When PB candidates are evaluated for PB performance, the use of a terminal-cross or a univariate model does not substantially change the accuracy of their EBV. However, if the aim is to evaluate the PB candidates for CB performance, accounting for both PB and CB information greatly contributes to improving the theoretical accuracy of the EBV, especially if the selection candidates have their own phenotypes (Table 7). The latter would be advantageous for traits that are routinely evaluated in the nucleus of selection. Nonetheless, if the candidate is not phenotyped, the accuracy of the EBV obtained with the GEN model was still slightly higher than with the univariate model (Table 7). This could be of interest for genetic evaluation of traits that are not directly recorded on candidates but only on a few relatives in test stations, e.g. meat quality and carcass traits.

**Table 7:** Mean accuracy of EBV for purebred and crossbred performance obtained using single-step terminal-cross models and its difference from the mean accuracy (in parentheses) obtained using pedigree-based terminal-cross models (in parentheses)

Trait	PB performance			CB performance		
	Sires	Phenotyped candidates	Unphenotyped candidates	Sires	Phenotyped candidates	Unphenotyped candidates
ADG	0.660 (0.110)	0.574 (0.026)	0.420 (0.059)	0.697 (0.046)	0.509 (0.181)	0.413 (0.084)
FCR	0.732 (0.113)	0.659 (0.020)	0.489 (0.054)	0.745 (0.065)	0.614 (0.259)	0.477 (0.121)
LM	0.716 (0.046)	0.696 (0.007)	0.490 (0.023)	0.724 (0.041)	0.535 (0.185)	0.434 (0.084)
pH	0.745 (0.133)	0.638 (0.026)	0.484 (0.068)	0.750 (0.080)	0.624 (0.273)	0.4807 (0.130)
DL	0.768 (0.046)	0.768 (0.005)	0.535 (0.024)	0.741 (0.119)	0.678 (0.371)	0.493 (0.185)
IMF	0.688 (0.105)	0.625 (0.019)	0.450 (0.053)	0.730 (0.040)	0.547 (0.184)	0.442 (0.078)

*PB* purebred, *CB* crossbred, *ADG* growth rate between end and beginning of the control period, *FCR* feed conversion ratio, *LM* % of lean meat, *pH* pH *longissimus dorsi*, *DL* drip loss, *IMF* intramuscular fat

### Predictive ability

Table 8 shows the average correlation between the predicted and yet-to-be observed PB and CB adjusted phenotypes obtained with sixfold cross-validation for all models and traits. Accounting for genomic information increased the predictive ability compared to using only pedigree-based relationships for all traits (predictive correlations for PB and CB performances were respectively 0.02–0.11 and 0.03–0.05 higher with the GEN model than with the PED model), except for ADG, for which no increase in predictive ability was observed. Nevertheless, the joint analysis of PB and CB performance in a single-step terminal-cross model did not substantially increase the predictive ability compared to single–step univariate analyses. The predictive ability of sire EBV estimated with the univariate single-step model for PB performance to predict CB performance was equal to 0.17, 0.18, 0.20, 0.08, 0.12 and 0.12 for ADG, FCR, LM, pH, DL and IMF, respectively. These results indicate that EBV estimated with the GEN_UNI model for PB performance predicted CB performance with the same accuracy as the models that accounted for CB performance (i.e. GEN and GEN_UNIC CB models). This could be due to the high estimated genetic correlations between PB and CB performance for the analyzed traits. It would be interesting in a future study to test the predictive ability of the GEN model when PB and CB performance are less genetically correlated (e.g. when purebreds are raised in selection nucleus and crossbreds under field conditions, or for traits that are strongly influenced by non-additive genetic effects).

**Table 8:** Average correlation between predicted and yet-to-be observed purebred and crossbred adjusted phenotypes obtained with a sixfold cross-validation

Model	Trait	PB phenotype	CB phenotype
PED	ADG	0.208	0.159
	FCR	0.139	0.166
	LM	0.122	0.148
	Ph	0.051	0.035
	DL	0.260	0.128
	IMF	0.178	0.086
GEN	ADG	0.204	0.191
	FCR	0.180	0.201
	LM	0.245	0.175
	pH	0.075	0.087
	DL	0.374	0.134
	IMF	0.225	0.072
GEN_UNI	ADG	0.195	0.191
	FCR	0.179	0.210
	LM	0.244	0.144
	pH	0.150	0.051
	DL	0.368	0.057
	IMF	0.236	0.051

*PB* purebred, *CB* crossbred, *ADG* growth rate between end and beginning of the control period, *FCR* feed conversion ratio, *LM* % of lean meat, *pH* pH *longissimus dorsi*, *DL* drip loss, *IMF* intramuscular fat

### Practical implications

Routine genetic evaluations in Piétrain pigs (as well as in other PB pig sire lines) are usually performed with pedigree-based BLUP using phenotypes of selection candidates and, for some traits, using phenotypes recorded on relatives obtained from test stations. Although selection is implemented within PB lines, the ultimate aim is to improve CB performance under field conditions. If the genetic correlations between PB and CB performance differ from 1, incorporating CB and genomic information into the genetic evaluation of the PB lines can contribute to increase genetic gains.

To the best of our knowledge, this is the first implementation of a single-step terminal-cross model using PB sire genotypes to model CB performance and its application to real data for a wide range of traits. Christensen et al. [13] developed a more complex single-step method for the genomic evaluation of PB and CB performance. Their model makes full use of genotypes on CB individuals and therefore accounts for the exact contribution of alleles of the sire and the dam to a given CB performance. Further research should compare their model with the GEN model with respect to their ability to predict new data, i.e. candidates to selection without phenotypic records. It is also necessary to determine whether the extra genetic progress achieved with our model overcomes the additional expenses of its implementation under commercial conditions, which would require substantial organizational changes in the breeding scheme, such as collecting phenotypes on the CB offspring, i.e. piglet production records collected from multiple commercial farms, genotyping selection candidates and tracing the pedigree to connect crossbreds with purebreds. Availability of the PB phenotype would be advantageous. Nonetheless, the main advantage of the GEN model is that CB genotypes would not be needed, which would limit extra expenses, and the dam contribution could be accounted for in the model as a permanent environmental effect.

## Conclusions

We proposed and applied on real data a single-step terminal-cross model that accounts for genomic information on PB individuals and uses CB performance to estimate genetic parameters of several production and quality traits in pigs. Accounting for PB and CB information, along with genomic information, improves the theoretical accuracy of genetic evaluations in breeding programs that are based on crossbreeding. Including genomic information increased predictive abilities compared to using pedigree information only, but the single-step terminal-cross model did not outperform the predictive performance of univariate single-step models for PB and CB performance. The implementation of the proposed single-step terminal-cross model is straightforward with available software but its use under field conditions needs to be further addressed in terms of predictive ability, genetic progress achieved, and costs.
